# What's the effect of the implementation of general practitioner cooperatives on caseload? Prospective intervention study on primary and secondary care

**DOI:** 10.1186/1472-6963-10-222

**Published:** 2010-07-30

**Authors:** Hilde Philips, Roy Remmen, Paul Van Royen, Marc Teblick, Leo Geudens, Marc Bronckaers, Herman Meeuwis

**Affiliations:** 1 University of Antwerp, Department of General Practice, Interdisciplinary Care and Geriatrics, Universiteitsplein 1, Gebouw R, 3de verd. B-2610 Wilrijk, Belgium; 2 Huisartsenvereniging Regio Turnhout (HVRT), Campus Blairon 410, B-2300 Turnhout, Belgium; 3 Algemeen Ziekenhuis Sint Jozef, Steenweg op Merksplas 44, B-2300 Turnhout, Belgium; 4 Sint Elisabeth Ziekenhuis, Rubensstraat 166, B-2300 Turnhout, Belgium

## Abstract

**Background:**

Out-of-hours care in the primary care setting is rapidly changing and evolving towards general practitioner 'cooperatives' (GPC). GPCs already exist in the Netherlands, the United Kingdom and Scandinavia, all countries with strong general practice, including gatekeepers' role. This intervention study reports the use and caseload of out-of-hours care before and after implementation of a GPC in a well subscribed region in a country with an open access health care system and no gatekeepers' role for general practice.

**Methods:**

We used a prospective before/after interventional study design. The intervention was the implementation of a GPC.

**Results:**

One year after the implementation of a GPC, the number of patient contacts in the intervention region significantly increased at the GPC (OR: 1.645; 95% CI: 1.439-1.880), while there were no significant changes in patient contacts at the Emergency Department (ED) or in other regions where a simultaneous registration was performed. Although home visits decreased in all general practitioner registrations, the difference was more pronounced in the intervention region (intervention region: OR: 0.515; 95% CI: 0.411-0.646, other regions: OR: 0.743; 95% CI: 0.608-0.908). At the ED we observed a decrease in the number of trauma cases (OR: 0.789; 95% CI: 0.648-0.960) and of patients who came to hospital by ambulance (OR: 0.687; 95% CI: 0.565-0.836).

**Conclusions:**

One year after its implementation more people seek help at the GPC, while the number of contacts at the ED remains the same. The most prominent changes in caseload are found in the trauma cases. Establishing a GPC in an open health care system, might redirect some patients with particular medical problems to primary care. This could lead to a lowering of costs or a more cost-effective out of hours care, but further research should focus on effective usage to divert patient flows and on quality and outcome of care.

## Background

From the nineties, general practitioner cooperatives (GPC) were established in many European countries, as a new alternative for the organisation of out-of-hours medical care by general practitioners. Various models exist across health care models. Although we do not have a clear-cut definition of 'appropriate use' or, inappropriate use' of the ED, it has been argued that many medical problems presented at the ED could easily be managed in a primary care setting [1,2]. Many studies report overuse of the ED for primary care medical problems [3-11]. One objective therefore may be to redirect patients from secondary care to primary care [12]. This could be a cheaper alternative and may in turn preserve funds dedicated to health care.

Common objectives for implementation of GPC are to relieve the burden of being on call for GPs, caused by a shortage of GPs, the increasing workload and dissatisfaction among GPs because of the lack of separation between work and private life [13]. Until now, most studies compared differences between different models of services, e.g. concerning accessibility and location [14-17].

Only a few studies assessed the impact of an intervention at the level of the implementation of a GPC in a before/after design [13,18].

The focus of the present study is on the patient fluxes to primary and secondary care during out-of -hours services. This study was performed in Belgium, which shows free access to primary and secondary care, no gatekeepers' role for the general practitioner (the GP does not control referral or access to secondary care) and a fee for service system. Large-scale GPC are being introduced from 2003 onwards. We asses the research question: What is the impact of the implementation of a general practitioner cooperative on the use and caseload of out-of-hours primary and secondary care?

## Methods

We used a prospective before/after study design. The intervention was the implementation of a GPC in the Turnhout region of Belgium.

### Intervention region

One of the characteristics of Belgian health care is the free access in primary care as well as in secondary and tertiary care. Also during out-of-hours, patients have a free choice between the general practitioner on call or the ED of a hospital. They do not need any referral by a physician. There is no need for any telephone contact before turn in to either one service. GPs are obliged to offer continuity of care. Recently GPs choose to implement GPCs (as in our intervention region in Turnhout) aiming a decrease in inappropriate use of EDs. Before the implementation of the GPC, GPs worked in a rota arrangement and organised out-of-hours care from their own practices. Patients had to inform themselves which GP was available and where his practice was located; they had the possibility to go to the doctors' practice or to ask the doctor on a home visit. There was no telephone triage. No consultation over the telephone was performed. The GPC re-organised all of the 100 GPs in that region and centralised the location for out-of-hours primary health care in one centrally located practice. That way the GPC is more accessible and recognisable for the whole region, in contrast to the former situation when the GP on call was at a different location at every turn. The GPC is open from Saturday 8 am until Monday 8 am and on public holidays, but not during weekdays. Three GPs are continuously present at the GPC for consultations; two other GPs are responsible for the home visits. The GPC is well-equipped, not only for dealing with urgent medical problems but also to be able to handle wound care and minor trauma. GPs on call have to report figures of all patient contacts to the local GP organisation. The Turnhout region shows tight boundaries, meaning that all patients living in Turnhout region seek help in one of the two hospitals with ED facilities in the city centre or at the GP service. More than 98% of the referrals by physicians in this region, are made to these two hospitals [19].

### Seasonal effects

To allow the monitoring of other effects on caseload (seasonal epidemiologic changes, awareness of changing primary health care during out-of-hours, changing payment systems at the ED), we used two regions to function as 'control' groups. These were chosen in regions where no GPC existed and where no GPC was planned; this is the case in suburbs of two other large cities (Ghent and Antwerp).

In these regions, GPs still work on an individual base, out of their own practice in a rota arrangement during weekends and public holidays. The regional union of GPs decides upon the sequence of the on-call rota on a regular basis. In this study, the GPs on call had to be able to register patient contacts and most of them used electronic patient records for this purpose. GPs that did not use electronic patient records filled out a registration form, which were collected by the research assistant.

**Figure 1:**
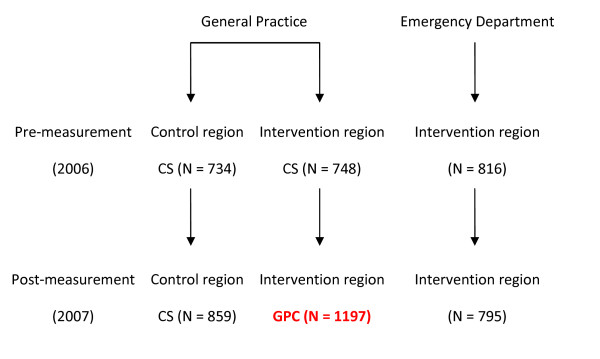
**Pre- and post-measurement in general practice and emergency departments**. (CS: GP out of hours care using the Classical System, DS: GP out-of-hours care at the new Deputising Service)

Due to vague boundaries of the catchment areas of the hospitals in these other regions, enrolling patients at the ED would not provide us with valid information about case-load. (fig 1) The data of ED in this region were not used. The GPs were included for descriptive reasons, to estimate the changes over the same time period.

### Instrument

We introduced identical forms for the patient registration at the ED and for the GPs on call. These forms could easily be filled out by the staff at the ED as well as by the GP on call. We piloted two months before the actual registration started and some small changes (lay-out, formulation of questions) in consensus with the users (GPs and the ED) were adapted.

Our first data collection was performed in 2006 (during two months, data from 9 weekends), two months before implementation of the GPC, and in 2007 (during the same two months, data from 9 weekends), one year after starting the GPC. For the second registration at the GPC, an electronic medical record system was used.

Besides patient characteristics (age, sex and zip-code), date, hour and type of the patient contact, we also registered clinical data: i.e. reason for encounter (RFE), physical examination, technical investigations and diagnosis.

To optimise participation, a research assistant contacted the GPs on call on a weekly basis in case of any problems filling out the forms. The EDs were visited on a monthly basis to collect the data and provide registration forms. Telephone calls to key persons on a regular basis also stimulated participation. To assess workload in the other participating regions, all routine patient data was collected using an MS Access registration tool for GP out-of-hours care. Validity of the first measurement data was checked by the number of registrations during the same period the year before our study.

### Data collection and analysis

We studied all patient contacts at the ED in both hospitals and with the GPs on call in the intervention region. During the same period we also registered the patient contacts of GPs on call in the other two regions. Data collection was performed starting from Saturday 8 a.m. until Monday 8 a.m. Personal data of the patients was removed from the records. Subsequently all registration forms were coded for 'reason for encounter' (RFE) and 'diagnose/diagnostic hypothesis' using the International Classification of Primary Care, 2^nd ^edition (ICPC2) by the first author. When two or more complaints or diagnoses were mentioned, the one interpreted as the most important was used. For instance a patient presenting himself with fever and diarrhoea was registered as having diarrhoea to be as specific as possible. After coding, the forms were enrolled in an MS Access or MS Excel database.

We used SPSS 14.0 for final data collection and analysis. We used uni-variant analysis with odds ratios and 95% confidence interval where applicable. We used Chi^2^-tests when comparing 2 or more nominal variables. Mann Whitney tests were used for comparison of mean ages. For several analyses we categorized age data in 5 categories (<12 y, 12-19 y, 20-64 y, 65-79 y, >79 y).

#### Approval of the ethical committee

Approval of the ethical committee was given by both hospitals and by the ethical committee of the Universitair Ziekenhuis Antwerpen (Academic Hospital of Antwerp) (ref nr: EC/PC/kv/2008).

## Results

### Workload

During both registration periods all 5149 patient contacts were included in the study: 2298 during the pre-measurement period (2006) and 2851 during the post-measurement period (2007). Patients enrolled in the registration of the intervention region were included based on zip-code. In the intervention region, cases belonging to other zip-codes were excluded from the database, this was necessary to compare pre- and post-measurement data.

In the intervention region, the number of patient contacts at the GPC during the second period increased significantly compared to the contacts with the GP on call in the first period (both including consultation and home visits) (OR: 1.645; 95% CI: 1.439-1.880). Although the total number of GP contacts in the other regions also increased, the difference was significantly larger in the intervention region (OR: 1.370; 95% CI: 1.198-1.565). The patient contacts at the ED did not change significantly over the same period. (fig 1)

### Patient characteristics

#### Age

Using the Mann-Whitney Test, there was a significant difference in mean age of the patients between the GP intervention group and the other GP groups, which persisted from the pre-measurement to the post-measurement (p < 0.01). The mean ages were respectively 37.2 y and 36.2 y in the intervention region, whereas it was 44.0 y and 40.8 y respectively in the other regions. We did not find significant shifts in mean age concerning GP or ED choice in the intervention region.

#### Sex

In general, more women seek help at the primary care settings, whereas men represent the majority of ED visitors (pre- measurement chi^2 ^= 36.087, p < 0.01; post-measurement chi^2 ^= 25.260, p < 0.01). We found no significant differences within the groups between the pre- and post-measurement.

### Type of contact

In table 1 we describe the evolution of the type of contact at the ED. There was no significant difference in patients who came on 'self-referral', between the pre- and post-measurement. We found significant changes between pre- and post measurement in the group of patients who were referred by a physician (general practitioner or specialist) or who came in by ambulance. The first group significantly increased (OR = 1.446; 95% CI: 1.196-1.749), whereas the second significantly decreased (OR = 0.687; 95%CI: 0.565-0.836).

**Table 1:** Changes in the number of the different types of contact at the emergency department between pre-and post measurement.

	Emergency Department			
	**Self referral**	**Referral by a physician**	**By ambulance**	**Total**

**Pre-measurement**	587 (72%)	94 (12%)	134 (16%)	**815**

**Post-measurement**	578 (73%)	127 (16%)*	86 (11%)**	**791**

Total	**1165**	**221**	**220**	**1606**

* significant increase (p < 0.05), ** significant decrease (p < 0.05)

The type of contact with the GPs also changed. The absolute number of home visits remained the same but relatively decreased compared to the consultations (intervention region OR = 0.515; 95%CI: 0.411-0.646 and other regions OR = 0.743; 95%CI: 0.608-0.908). In the other regions the relative number of home visits also decreased significantly, but not as prominent. (table 2)

**Table 2:** Evolution of the type of GP contacts.

	Type of GP contact			
		**Consultation (%)**	**Home visit (%)**	**Total amount of contacts**

**Intervention region**	Pre-measurement	520 (73%)	194 (27%)	**714**

	Post-measurement	1004 (84%)*	193 (16%)**	**1197**

	**total**	**1524**	**387**	**1911**

**Other regions**	Pre-measurement	404 (55%)	330 (45%)	**734**

	Post-measurement	529 (62%)*	321 (38%)**	**850**

	**total**	**933**	**651**	**1584**

* significant increase (p < 0.05), ** significant decrease (p < 0.05)

When we consider age in 5 categories we find significant changes over time in the type of GP contact. In the intervention region there is a significant shift from home visits to consultations for all age categories except for the '+79 years of age'. In the other regions, a similar shift was only found in the youngest age category, while the other categories did not change significantly. (table 3)

**Table 3:** Odds ratio's for 5 age-categories, concerning differences in type of GP contact in the pre-and post-measurement.

	Intervention region Consultation/home visit Post-measurement/pre-measurement	other regions Consultation/home visit Post-measurement/pre-measurement
**<12 y**	**OR: 5.924** **95% CI: 1.178-29.800**	**OR: 4.714** **95% CI: 1.845-12.044**

**12-19 y**	**OR: 5.886** **95% CI: 1.033-33.538**	OR: 1.056 95% CI: 0.245-4.540

**20-64 y**	**OR: 1.838** **95% CI: 1.313-2.571**	OR: 1.291 95% CI: 0.807-2.065

**65-79**	**OR: 1.930** **95% CI: 1.045-3.565**	OR: 2.187 95% CI: 0.692-6.910

**>80 y**	OR: 1.875 95% CI: 0.618-5.690	OR: 2.459 95% CI: 0.297-20.340

Significant differences are represented in bold.

### Case load using ICPC2 headings

All patient contacts were coded by ICPC2. For some ICPC headings significant differences between the pre- and post-measurements exist.

#### Reason for encounter (RFE)

For both, GP and ED, the most frequently used ICPC2-headings were: A (general and unspecified) (27.2%), D (digestive) (14.9%) en R (respiratory) (14.4%).

Of all the GP patient contacts the 3 most used ICPC2-headings were: R (respiratory)(18.5%), A (general and unspecified) (18.2%) and D (digestive) (17.5%). At the ED, the 'top 3' was: A (general and unspecified) (47.2%), S (skin) (10.3%) and L (musculoskeletal) (9.4%).

Over time, the ICPC2-heading, 'K' (circulatory), increased significantly at the ED. (OR: 1.743; 95% CI: 1.006-3.022) An analogue increase was found in ICPC2-headings, 'P' (psychological problems) (OR: 1.971; 95% CI: 1.086-3.579) and, 'L' (musculoskeletal) (OR: 1.971; 95% CI: 1.086-3.579).

We observe for RFE 'trauma-related complaints' (A80, A81 and A84) a significant decrease at the ED (table 4). Although the major part of people with trauma prefers ED, the case load at the GPC almost doubled (but not significantly) for these ICPC codes.

**Table 4:** proportional differences in case load of 'trauma related complaints' in the reason for encounter (RFE).

	Pre-measurement	Post-measurement	
**GP other regions**	36 (7.4%)	45 (9.1%)	OR: 0.993 95%CI: 0.595-1.463

**GP intervention region**	54 (11.1%)	108 (21.9%)	OR: 0.786 95%CI: 0.559-1.104

**ED**	397 (81.5%)	340 (69.0%)	**OR: 0.789;** **95%CI: 0.648-0.960**

Total	**487**	**493**	

Significant differences are represented in bold.

#### Diagnosis

The top 3 of diagnostic ICPC2-headings in the entire database (GP and ED) were: R (respiratory) (19.2%), L (musculoskeletal) (17.5%) and S (skin) (15.8%). For the overall GP patient contacts we found: R (respiratory) (22.9%), D (digestive) (15.5%) and L (musculoskeletal) (13.7%). At the ED, the top 3 percentages are: L (musculoskeletal) (30.2%), S (skin) (28.6%) and R (respiratory) (6.6%). Here again, few headings differ between the pre- and the post-measurement.

ICPC2-heading, 'D' (digestive) decreased significantly in the intervention region at the GPC (OR: 0.748; 95% CI: 0.577-0.971). Also ICPC2-heading P (psychological problems) decreased at the GPC in the post-measurement (OR: 0.424; 95% CI: 0.241-0.747). There were no significant differences in these headings in the other regions or at the ED.

At the ED the total amount of cases with the diagnosis in ICPC2-heading, 'S' (skin) or, 'L' (musculoskeletal) significantly decreased (OR: 0.578; 95% CI: 0.470-0.711), while there was no difference in the group of GPs, neither in the control, nor in the other regions. (table 5)

**Table 5:** Evolution of the case-load of cases with ICPC2 heading, 'L' or, 'S' in the diagnosis at the ED (p < 0.01).

	Pre-measurement	Post-measurement	Total
**Diagnoses** **ICPC2-heading S (skin) or L (musculoskeletal)**	342 (41.9%)	234 (29.4%)	**576**

**Other diagnoses**	474 (58.1%)	561 (70.6%)	**1035**

	**816**	**795**	**1611**

### Technical examinations

We assessed the number of technical examinations and used all cases where any technical examination was mentioned (blood- or urine analysis, swabs taken for culture, radiology (RX, CT, echo-graph, ECG)). Either the handling physician performed the examination himself or referred the patient for further technical examination. At the ED more than 60% of the patients received at least one technical examination, whereas the highest percentage in the GP groups was 5.6%.

## Discussion

To our knowledge, this study is the first to report the results of the implementation of a new GPC in a open access health care system. Caseload of the GP were doubled while there was no significant decrease of patient turnover at the ED. We also describe changes in patient contacts; consultations, home visits and ICPC2 codes for RFE and diagnosis.

We simultaneously collected data at GP services in other regions, where no GPC was established. Although not completely matched and lacking data of ED in the other regions, this methodology is probably the most feasible design to study changes in caseload when establishing a GPC. In the original study design we considered a time series study over 3 years time. However, due to changing software program at one of the hospitals, we were not able to collect comparable data during the third year. Therefore this design was not feasible.

In Belgium, all patients have free access and free choice during out-of-hours between the GP on call as well as to the ED of a hospital. GPs do not have a gatekeepers' role and entrance to health care is possible without referral by a physician or prior telephone contact. The possibility of a telephone consult or treatment by a practice nurse, as it is known in the Netherlands for instance, does not exist. In most regions, there are no defined regional catchment areas. Patients can easily seek help in a neighbouring village or city.

We chose Turnhout region as our study domain. This city has a well-defined catchment area, meaning that GPs as well as both hospitals cover the same region with negligible overlap with neighbouring regions. This enabled us to obtain a valid view on caseload at the GP and the ED. We included other regions in the neighbourhood of the cities of Ghent and Antwerp to have some account for changes like seasonal influences on epidemic changes or changes in patients' awareness of the use of out-of-hours services. Unfortunately including a control region for the ED was not feasible, because regions with tight boundaries are scarce. Secondly, there were (at the time of our study) no uniform information technology systems at the EDs in hospitals in Belgium. Similar to former research, we observed an increase of patient contacts at the GPC over a one year period [12,18,20,21]. However, in contrast with the studies performed in the Netherlands and the UK, we did not observe a significant decrease in patient numbers at the ED. This may be explained by the free access in the health care system in Belgium. The GPC was implemented without any changes or restrictions in accessibility to the ED. Moreover, the use of a service may be driven by the availability of this service, which is called the, push-strategy' [22-24]. Although in our study, the number of patients seeking help at the ED after referral by a physician increased, the number of self-referrals stayed the same. This suggests that patients who want to seek help at the ED without a referral, do not change their behaviour because of the presence of a GPC. On the other hand, there was a significant decrease in the number of patients who came to the ED by ambulance, which (in this country) can be called without any referral by a doctor. (table 1) Possibly, the presence of a GPC could lead to more efficient use of ambulances by creating an accessible and recognisable alternative when people are anxious or worried.

Currently there is a trend in this country, decreasing the share of home visits also during normal working hours [21,25]. In this study, this effect also occurs during out-of-hours and seems to be accelerated after implementation of a GPC. The decrease of home-visits was observed for all age categories, except for the very elderly. Home visits are necessary for this age group because of diminished mobility and are also the strength of general practice care [26]. The amount of home visits to the very elderly does not change significantly after establishing a GPC. This might indicate that equity for the elderly is also accomplished at the GPC.

There is a significant decrease at the ED covering RFE on circulatory (K) and psychological (P) problems. On the other hand digestive (D) and psychological (P) diagnosis decreased at the GPC. We have no explanation for this. We also observed a significant decrease in 'trauma' cases at the ED, whereas the contacts with wound- or trauma related diagnoses ('L' and, 'S' diagnoses) slightly increased at the GPC. We might hypothesise that the presence of the GPC lowers the threshold to seek medical advice from a GP, also for minor trauma. One of the aims of the GPC is dealing with minor trauma and wound-care by being well-equipped. Accessibility has improved due to the fixed, central and recognisable location of the GPC in the city. The results seem to affirm that patients tend to recognise the role of the GP in these types of medical problems.

In this study we found a large amount of technical examinations at the ED. We could expect lower costs when more trauma cases could be dealt with at the GPC. Future research is needed to study the difference in costs due to a possible difference in assessment of the same medical problem at the GPC and the ED. Also outcome data in terms of health benefit should be investigated between services.

More is needed to realize effective shifts of patients from the ED to the primary care setting during out-of-hours services. A more explicit image of primary health care is needed, as stated in the latest WHO report [27]. Thanks to our former research in which we studied patients' preferences, we can confirm this need also in Belgium. In this specific health care system, centrally delivered information to patients about the tasks and skills of GPs, is necessary. A first-time contact of high-quality influences patient attitudes positively. From former research we know that people prefer a doctor who informs them about the illness and the treatment in a clear way. If this condition is met, patients tend to return to the service they are familiar with [28]. In the same subject we look out for the results of another study we performed in Belgium, using discrete choice analysis. This methodology is adopted from management studies and was already used in medical research by several authors [29,30].

The GPC is not available during weekdays. Therefore changing behaviour in patients might be more difficult. In future research, a comparison in patient choice during weekdays or weekends can clarify whether establishing a GPC during weekdays is a useful option. It certainly would clarify the role and organisation of out- of- hours healthcare for the users.

## Conclusions

Although we observed that starting a GPC does not immediately lead to patient fluxes away from the ED (total amount of patient contacts at the GPC increased while remaining the same at the ED), further research needs to be done to see if it does actually lead to better quality of care and patients satisfaction, with respect for equity.

## Competing interests

The authors declare that they have no competing interests.

## Authors' contributions

All authors read and approved the final manuscript. PH contributed to the study design, data gathering, analysis and writing the text. RR contributed to the study design, data gathering, analysis and reviewing the text. VRP contributed to the study design, analysis and reviewing the text. TM contributed to the study design, data gathering and reviewing the text. GL contributed to the study design, data gathering and reviewing the text. BM contributed to the study design, data gathering and reviewing the text. MH contributed to the study design, data gathering and reviewing the text.

## Pre-publication history

The pre-publication history for this paper can be accessed here:

http://www.biomedcentral.com/1472-6963/10/222/prepub
